# Predicting the current habitat refugia of Himalayan Musk deer (*Moschus chrysogaster*) across Nepal

**DOI:** 10.1002/ece3.10949

**Published:** 2024-02-16

**Authors:** Bijaya Dhami, Nar Bahadur Chhetri, Bijaya Neupane, Binaya Adhikari, Bijay Bashyal, Tek Maraseni, Tilak Thapamagar, Yogesh Dhakal, Aashish Tripathi, Narayan Prasad Koju

**Affiliations:** ^1^ Department of Biological Sciences University of Alberta Edmonton Alberta Canada; ^2^ IUCN/SSC Deer Specialist Group Gland Switzerland; ^3^ Division Forest Office Myagdi Nepal; ^4^ Institute of Forestry, Pokhara Campus Tribhuvan University Pokhara Nepal; ^5^ Department of Forest Sciences, Faculty of Agriculture and Forestry University of Helsinki Helsinki Finland; ^6^ Department of Biology University of Kentucky Lexington Kentucky USA; ^7^ Central Department of Environmental Science Tribhuvan University Kathmandu Nepal; ^8^ University of Southern Queensland Toowoomba Queensland Australia; ^9^ Himalayan Biodiversity Network Nepal Chitwan Nepal; ^10^ Ministry of Forests and Environment Kathmandu Nepal; ^11^ Center for Post Graduate Studies, Nepal Engineering College Pokhara University Bhaktapur Nepal

**Keywords:** anthropogenic variables, climate change impacts, habitat suitability modeling, Himalaya, predictive performance, species distribution models

## Abstract

Himalayan Musk deer, *Moschus chrysogaster* is widely distributed but one of the least studied species in Nepal. In this study, we compiled a total of 429 current presence points of direct observation of the species, pellets droppings, and hoofmarks based on field‐based surveys during 2018–2021 and periodic data held by the Department of National Park and Wildlife Conservation. We developed the species distribution model using an ensemble modeling approach. We used a combination of bioclimatic, anthropogenic, topographic, and vegetation‐related variables to predict the current suitable habitat for Himalayan Musk deer in Nepal. A total of 16 predictor variables were used for habitat suitability modeling after the multicollinearity test. The study shows that the 6973.76 km^2^ (5%) area of Nepal is highly suitable and 8387.11 km^2^ (6%) is moderately suitable for HMD. The distribution of HMD shows mainly by precipitation seasonality, precipitation of the warmest quarter, temperature ranges, distance to water bodies, anthropogenic variables, and land use and land cover change (LULC). The probability of occurrence is less in habitats with low forest cover. The response curves indicate that the probability of occurrence of HMD decreases with an increase in precipitation seasonality and remains constant with an increase in precipitation of the warmest quarter. Thus, the fortune of the species distribution will be limited by anthropogenic factors like poaching, hunting, habitat fragmentation and habitat degradation, and long‐term forces of climate change.

## Graphical abstract

Himalayan Musk deer, *Moschus chrysogaster* (HMD) is widely distributed but one of the least studied species in Nepal. We used a combination of bioclimatic, anthropogenic, topographic, and vegetation‐related variables to predict the current suitable habitat for HMD in Nepal using 16 predictor variables for habitat suitability modeling after the multicollinearity test. We explored the ensemble model outperformed single algorithm models in terms of predictive performance (0.966) showing 6973.76 km^2^ (5%) area of Nepal is highly suitable and 8387.11 km^2^ (6%) is moderately suitable for HMD.

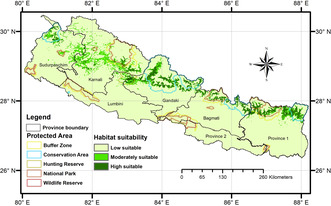

## 1 INTRODUCTION

Himalayan Musk deer (*Moschus chrysogaster*) (hereby HMD) is a shy solidary crepuscular high‐altitude mammal species belonging to the family Moschidae and regarded as the indicator of the integrated Himalayan ecosystem (Lamsal et al., 2018). It is a globally threatened species listed as “Endangered” in IUCN Red Data Book (Harris, 2016) and as per national concern, it is an endangered species (Amin et al., 2018; Jnawali et al., 2011; Timmins & Duckworth, 2015), listed in Appendix I of CITES (Amin et al., 2018) and Protected species listed by National Park and Wildlife Conservation Act‐1973 (DNPWC, 2013; Lamsal et al., 2018). There are seven species of Musk deer, namely, *Moschus moschiferus*, *M*. *fuscus*, *M*. *berezovskii*, *M*. *sifanicus*, *M*. *cupreus*, *M*. *chrysogaster*, and *M*. *leucogaster*. Among these species, *M*. *chrysogaster*, *M*. *cupreus*, *M*. *fuscus*, and *M*. *leucogaster* are found in Nepal (Green, 1986; Koju, Bashyal, & Shah, 2021; Lamsal et al., 2018; Singh, Gautam, et al., 2020; Singh, Mainali, et al., 2020). *M*. *chrysogaster*, *M*. *fuscus*, and *M*. *leucogaster* inhabit in similar habitat, genetically they have very close relation with low divergences but listed separate species based on morphological feature only (Singh, Gautam, et al., 2020; Singh, Mainali, et al., 2020; Su et al., 1999). Among six types of deer in Nepal, HMD is one of them, which is found in the Himalayan alpine forest habitats between 2200 and 4300 m elevations (Lamsal et al., 2018). HMD is solitary and territorial species that is usually a concentrate feeder but can adapt to poorer diets in low‐quality habitats (Green, 1986).

The Musk deer species are distributed through at least 13 countries in South Asia, East Asia, Southeast Asia, and Eastern Russia (Xiuxiang et al., 2006). The HMD species is native to Nepal, China, India, and Bhutan; however, it has been recorded in Pakistan, Myanmar, and Afghanistan as well. In Nepal, it is distributed throughout the Himalayan region with 5815.08 km^2^ of area inside protected areas (Aryal & Subedi, 2011). Among the 20 protected areas of the country, it is distributed majorly in Api Nampa Conservation Area (ANCA), Khaptad National Park (KNP), Rara National Park (RNP), Shey Phoksundo National Park (SPNP), Dhorpatan Hunting Reserve (DHR), Annapurna Conservation Area (ACA), Manaslu Conservation Area (MCA), Langtang National Park (LNP), Makalu Barun National Park (MBNP), and Kanchanjunga Conservation Area (KCA) (Amin et al., 2018; Green, 1986; Harris, 2016; Jnawali et al., 2011; Koju, Bashyal, & Shah, 2021; Lamsal et al., 2018; Neupane et al., 2021; Singh, Gautam, et al., 2020; Singh, Mainali, et al., 2020).

The distribution of musk deer is influenced by several factors including habitat, climate, and anthropogenic aspects (Singh, Gautam, et al., 2020; Singh, Mainali, et al., 2020). Studies have reported that climatic variables have greatly contributed to the distribution of musk deer in the Nepalese Himalayas (Lamsal et al., 2018; Singh, Gautam, et al., 2020; Singh, Mainali, et al., 2020). Among the climatic factors, precipitation was recognized as the most important factor for predicting the habitat suitability of the species, on the suitable habitat was found in the higher precipitation areas (Khadka et al., 2017). Similarly, the temperature has also a prominent role in the habitat suitability of musk deer (Lamsal et al., 2018). The other important habitat variable is vegetation which is responsible for determining the habitat suitability of this species (Nandy et al., 2020). However, this vegetation growth or availability is positively associated with precipitation (Tiwari et al., 2017). Regarding the habitat variable distance to water sources, the probability of occurrence of musk deer decreased with the increase in distance to water sources (Thapamagar et al., 2021). Similarly, musk deer was found in the gentle slopes up to 20 degrees as mentioned by earlier studies (Aryal et al., 2010; Neupane et al., 2021). Regarding the anthropogenic factors, the musk deer usually avoids human activities and livestock grazing sites in human‐dominated landscapes (Thapamagar et al., 2021). However, there is a seasonal and temporary nature of settlements in the high Himalayas of Nepal, so the species might overlap with the settlements, particularly during the winter season when people move to the lowlands to avoid extreme cold weather (Nandy et al., 2020).

In species distribution modeling (SDM) or ecological niche modeling, the potential distribution of a species is explained by the relationship between the species and the surrounding ecological and environmental factors (Beery et al., 2021; Peterson & Soberón, 2012; Thuiller et al., 2009). SDM can be used as a conservation planning approach for threatened species by determining the species distribution range and ecological niche (Adhikari et al., 2019). Due to the presence of large data and multifaceted associations between species and ecological variables, the scope of computer algorithms for ecological niche modeling, habitat modeling, predictive habitat distribution modeling, and range mapping such as SDM has increased to solve the problem of ecologists and statisticians (Beery et al., 2021). Besides, SDM helps to envisage the effects of climate change on species, which is very essential to achieve the conservation goals of being aware of the species distribution (Forester et al., 2013; Raymond et al., 2020). Discrepancies among different SDMs create challenges in determining the optimal model choice (Elith et al., 2011; Elith & Leathwick, 2009; Renner & Warton, 2013). This becomes especially evident when models are employed to forecast species distribution in distinct scenarios, such as projecting it into varied geographic regions (Thuiller, 2004; Thuiller et al., 2009). The ensemble modeling approach offers a viable solution to navigate through these complexities. Through the ensemble method in SDM, several modeling techniques are assembled to improve the projecting performance (Hao et al., 2020). The temperature has been found as a significant variable in shaping the distribution of several Himalayan species (Elsen et al., 2017; Koju, Bashyal, & Shah, 2021; Koju, Chalise, & Kyes, 2021). So, the wildlife of higher elevations or mountainous regions is more vulnerable to the impacts of climate change (Aryal et al., 2016; Elsen et al., 2020). Change in the vegetation composition and shift in the vegetation range have been documented from different regions of the Himalayas: i.e. west (Lamsal et al., 2018), East (Manish et al., 2016), and Central (Chhetri & Cairns, 2015) which are major consequences of climate change. This anticipated climate change will alter the climatic niche and shift the geographical ranges of several faunal species in the future. For example, about 30% of snow leopard (*Panthera uncia*) living space is anticipated to be lost in the entire Himalayan area by 2050, of which 40% could vanish from Nepal (Forrest et al., 2012). Likewise, Aryal et al. (2016) anticipated diminished habitat for snow leopards and blue sheep (*Pseudois nayaur*) in Nepal in the future climate. All of these confirmations recommended that climatic change drives the species to modify their geographic distribution in each locale, including the Himalayas. With concern to HMD, despite the anthropogenic activities such as habitat loss, habitat degradation, and poaching being the major factors leading to the population decline of HMD (Harris, 2016; Jnawali et al., 2011; Neupane et al., 2021), the species are additionally affected by the effect of climate change with a fluctuating level of results over space and time (Lamsal et al., 2018; Van Gils et al., 2016), Thus, the conservation of such threatened and crucial species of Himalaya region is highly essential in the scenario of the projected increase in climate‐induced warming on those regions. This study aims to evaluate the appropriateness of the habitat for the endangered HMD species by examining various ecological and anthropogenic factors in their existing distribution areas. It is crucial to identify suitable habitats to enhance connectivity and ensure long‐term conservation efforts. We hypothesized that HMD might exhibit a preference for habitats near water sources while avoiding areas that have significant anthropogenic influence. Additionally, we anticipate that a substantial portion of potentially suitable habitat exists outside of the currently protected areas. By investigating these aspects, we aim to gain a comprehensive understanding of HMD's habitat requirements and contribute to effective conservation strategies.

## 2 MATERIALS AND METHODS

### 2.1 Study area

Nepal is a mountainous country that extends over 147,516 km^2^ in South Asia between the latitudes of 26°22′ to 30°27′ north and longitudes of 80°04′ to 88°12′ east. Because of the variability of climate and topography along a strong altitudinal gradient spanning from 60 to 8848 m above mean sea level, the country is endowed with extensive biodiversity (Bhattacharjee et al., 2017; Paudel et al., 2012). There are three major physiographic regions in Nepal: (1) lowland (Terai and Siwalik), (2) mid‐hills, and (3) high mountains (Shrestha & Aryal, 2011). The prevailing climate in the country is characterized by dry winters and hot summers (Karki et al., 2016). The average annual precipitation is 1768 mm and the annual mean temperature is 18°C (Shrestha et al., 2000). The high mountains, which are the preferred distribution range of HMD, cover 24% of the country's total geographical area and comprise two‐thirds of the country's PAs (Shrestha et al., 2010).

### 2.2 Data collection

#### 2.2.1 Presence data of HMD

**FIGURE 1:**
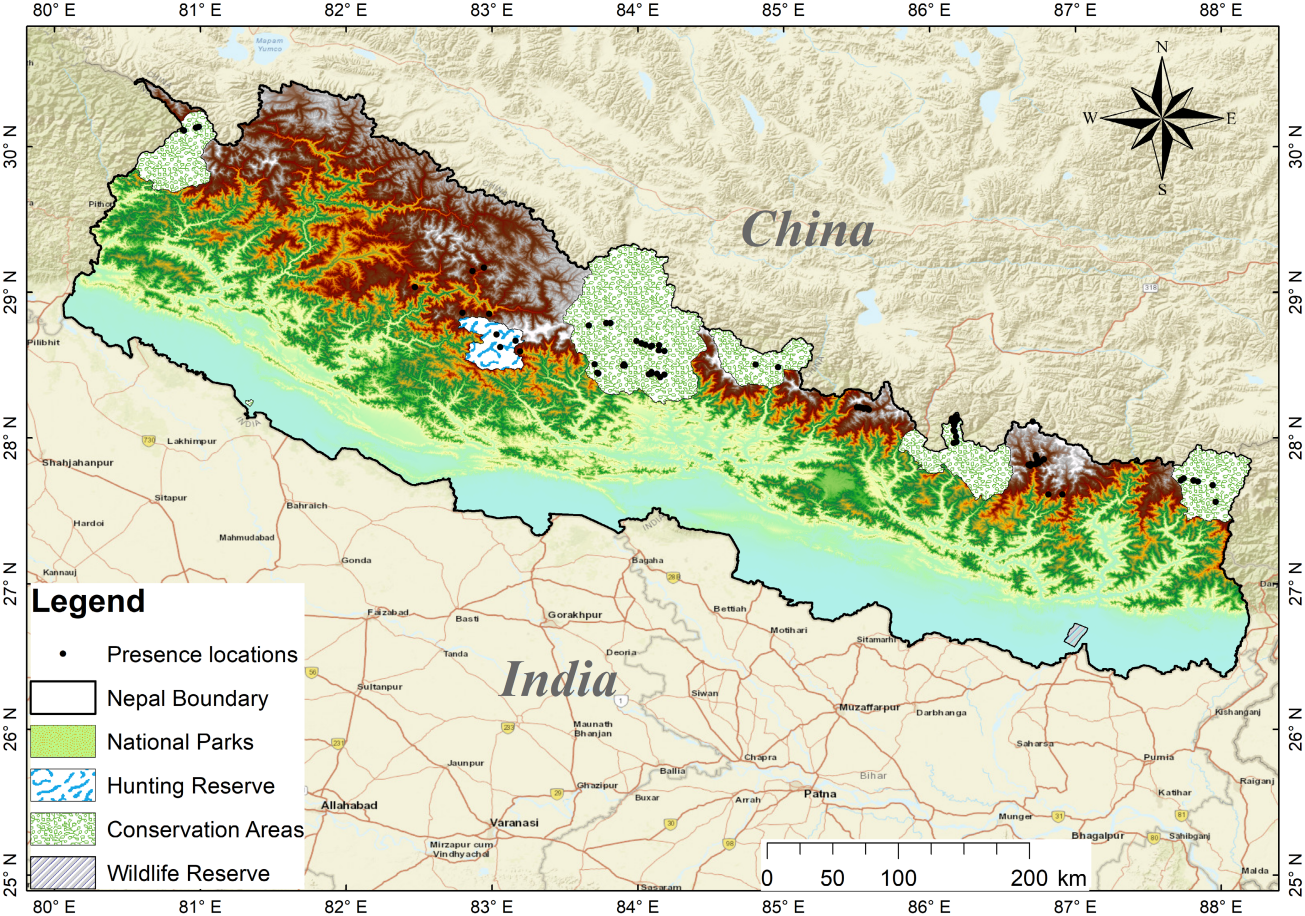
Map of study area showing the protected area types within Nepal and occurrence distribution throughout the study area.

We obtained the occurrence locations of HMD mainly from the field‐based surveys during 2018–2021 and Periodic data held by the Department of National Park and Wildlife Conservation of Nepal between 2018 and 2021. For field observations, we relied on direct sightings of the species, camera trapping as well as indirect sign sightings—pellets droppings and hoofmarks for determining the presence of HMD (Figure 1). Its pellets were differentiated from other sympatric deer species by their shape and size (Neupane et al., 2021). Within our pre‐defined study period, a total of 429 existing presence points of HMD were compiled. We used the spatial resolution of the environmental variables employed in this modeling was 1 km. Similarly, the SpThin package in R spatially attenuates the occurrence dataset ensuring that no two locations were inside a grid of 1 × 1 km (Aiello‐Lammens et al., 2015). Thus, only one presence point in each grid cell was used to minimize spatial autocorrelation and avert inflated measures of accuracy (Veloz, 2009). Spatial filtering also helps to improve model prediction performance by reducing the effects of sample bias (Boria et al., 2014). Following filtering procedures, a set of 346 spatially independent HMD presence locations were retained and used for modeling.

#### 2.2.2 Environmental variables

We used a combination of bioclimatic, anthropogenic, topographic, and vegetation‐related variables to predict the current suitable habitat for HMD in Nepal. Given that variable selection is regarded as a critical phase in SDM, an effort to incorporate important predictor variables (Araújo & Guisan, 2006) was used. Initially, we identified a set of 33 variables (Table 1) based on literature, which suggested those variables were important for the habitat suitability of HMD. We performed the multicollinearity test for the selected environmental variables and avoided the environmental variables with correlation coefficients >0.8 and variance inflation factor (VIF) >5, which helped to prevent our model overfitting. Finally, 16 variables were retained as predictor variables in habitat suitability modeling for HMD as suggested by Zeng et al. (2016).

**TABLE 1:** Sixteen environmental predictor variables (*) used for habitat suitability modeling.

Source	Category	Variable	Unit
WorldClim	Bioclimatic	BIO1 = Annual Mean Temperature BIO2 = Mean Diurnal Range (Mean of monthly (max temp − min temp)) * BIO3 = Isothermality (BIO2/BIO7) (×100) * BIO4 = Temperature Seasonality (standard deviation ×100) BIO5 = Max Temperature of Warmest Month BIO6 = Min Temperature of Coldest Month BIO7 = Temperature Annual Range (BIO5‐BIO6) BIO8 = Mean Temperature of Wettest Quarter BIO9 = Mean Temperature of Driest Quarter BIO10 = Mean Temperature of Warmest Quarter BIO11 = Mean Temperature of Coldest Quarter BIO12 = Annual Precipitation BIO13 = Precipitation of Wettest Month BIO14 = Precipitation of Driest Month * BIO15 = Precipitation Seasonality (Coefficient of Variation) * BIO16 = Precipitation of Wettest Quarter BIO17 = Precipitation of Driest Quarter BIO18 = Precipitation of Warmest Quarter * BIO19 = Precipitation of Coldest Quarter *	
USGS	Topographic	Elevation	km
		Aspect *	Degree
		Slope *	Degree
GEOFABRIK		Distance to water *	km
Landsat	Vegetation‐related	Mean EVI, Minimum EVI, Maximum EVI (Enhanced Vegetation Index)	Dimensionless
GFC		Forest *	Dimensionless
Department of Survey, Nepal	Anthropogenic	Distance to settlement *	km
GEOFABRIK		Distance to the motor road *	km
		Distance to path *	km
HUMDATA		Population density *	per km^2^
		Livestock density *	Per km^2^
ICIMOD		LULC *	km

#### 2.2.3 Bioclimatic variables

For spatial modeling, bioclimatic variables are widely used given that these variables are ecologically important and characterize annual trends, seasonality, and temperature and precipitation extremes (Hijmans, 2012; Hijmans et al., 2005). WorldClim‐Global Climate Data (www.worldclim.org/bioclim) was used to retrieve 19 bioclimatic variables (Fick & Hijmans, 2017). These data were retrieved in a grid format with a 1 km spatial resolution.

#### 2.2.4 Anthropogenic variables

Anthropogenic activities influencing the distribution of HMD have been identified as a threat (Syed & Ilyas, 2015; Thapamagar et al., 2021). Thus, these variables were also incorporated as predictor variables in our model. We used distance to a human path, distance to roads, distance to settlements, human population density, livestock density, and land use land cover data as the anthropogenic variables in the model. The data on the paths, roads, and buildings were extracted using Geofabrik's website (GEOFABRIK, 2022), while the data on settlements was obtained from the Nepalese Department of Survey, and a distance raster file was constructed using ArcGIS10.8.1 (ESRI, 2020). The data on land use and land cover change (2000–2019) was downloaded from the ICIMOD (ICIMOD, 2022). Similarly, data on human population density was obtained from the Humanitarian Data Exchange Dataset (HDX, 2022). Finally, the data on livestock density was obtained from the Ministry of Agricultural Development through Open Data Nepal (ODN, 2022).

#### 2.2.5 Topographic variables

Topographic variables such as elevation, slope, aspect, and distance to water sources have governed the habitat suitability of mega‐herbivores (Ghimire et al., 2019; Sharma et al., 2020). In our study, elevation, aspect, and slope data were generated using ArcMap 10.8.1 (ESRI, 2020) with a 1 km spatial resolution Digital Elevation Model (DEM) acquired from the United States Geological Survey database (USGS, 2022). Shapefiles including water source information were downloaded from the Geobabrik website (GEOFABRIK, 2022) and transformed into a distance raster file using ArcMap10.8.1 (ESRI, 2020).

#### 2.2.6 Vegetation related variables

One of the most important elements determining the distribution of herbivores like HMD is vegetation‐related variables (Gandiwa, 2014; Perea et al., 2015). The study collected four variables namely forest cover, minimum EVI, mean EVI, and maximum EVI. Forest cover was downloaded from Earth engine partner Appspot (Hansen et al., 2013). EVI time‐series data was obtained from Moderate Resolution Imaging Spectroradiometer (MODIS) (USGS, 2022). In the TIMESAT algorithm (Jönsson & Eklundh, 2004), the Savitzky–Golay filter was employed to smooth the data.

### 2.3 Data analysis

#### 2.3.1 Predicting the distribution of HMD

In the first step, a multicollinearity test was conducted among the 33 environmental variables. Variables displaying a correlation coefficient >.7 and a variance inflation factor >5 were excluded to mitigate the multicollinearity effect (Dormann et al., 2013). As a result, 16 predictor variables remained and were utilized for habitat suitability modeling (Table 1). We followed the overview, data, model, assessment, and prediction (ODMAP) method suggested by Zurell et al. (2020) to create habitat suitability models for HMD in Nepal. The utilization of ensemble maps in recent species distribution modeling (SDM) exercises has garnered considerable attention due to their demonstrated higher predicted accuracy (Hao et al., 2020). These maps are formed by merging multiple models constructed through diverse modeling approaches (Hao et al., 2019).

As a result, the habitat suitability model for HMD in Nepal was constructed using an ensemble modeling approach. The ensemble model was created in R (R Development Core Team, 2020) using the BIOMOD2 package (Thuiller et al., 2020). It was based on ten algorithms, including the artificial neural network (ANN), classification tree analysis (CTA), flexible discriminant analysis (FDA), generalized additive model (GAM), generalized boosting model (GBM), generalized linear model (GLM), multiple adaptive regression splines (MARS), maximum entropy (MAXENT), random forest (RF), and surface range envelope (SRE). Musk deer's presence and pseudo‐absence were split into two data sets: training (70%) and testing (30%). As recommended by Barbet‐Massin et al. (2012), we generated 10,000 pseudo‐absence points at random from the training dataset, allocating equal weights for the presence and pseudo‐absence datasets. To prevent random bias, we performed the pseudo‐absence generation three times. This modeling produced 90 model runs in all, including ten methods, three pseudo‐absence selection runs, and three evaluation runs. Both area under the receiver operating characteristics (ROC) curve, often called the area under the curve (AUC) (Lobo et al., 2008) and true skill statistics (TSS) (Allouche et al., 2006), are widely used measures to evaluate predictive performance (Thuiller et al., 2009). Despite being widely used as a measure for model evaluation, AUC is criticized for its shortcomings (Lobo et al., 2008). Hence, the predictive performance of our model was evaluated using TSS criteria ranging between −1 and +1. To construct an ensemble model through a weighted mean strategy, all models having a TSS value >0.6 (Marmion et al., 2009) were selected. Three models (GBM, MaxEnt, and RF) have TSS value greater than 0.6 and hence we selected them to develop a weighted mean ensemble approach.

Secondly, we also analyzed the data with Maxent only model, to observe whether the parameter tuned maxent only model performs better compared to the ensemble model approach. The ENMeval package (Kass et al., 2021) in the R programming language was employed to optimize the MaxEnt model. In this study, a comprehensive evaluation was conducted on a set of 48 models. These models were created by considering various combinations of five feature classes, namely, linear (L), quadratic (Q), hinge (H), product (P), and threshold (T). Additionally, the models were assessed using eight alternative regularization multiplier (RM) values ranging from 0.5 to 4, with an interval of 0.5. A total of 48 different combinations of parameters were evaluated. The best‐fit model was determined by employing the Akaike information criterion (AIC), a 5% training omission rate (OR5), and the comparison of AUC values (Dhami et al., 2023; Mao et al., 2022; Steen et al., 2019). Following the process of model optimization, the model that was selected as the best fit model had the characteristic class (Feature Class) LH, a value of RM equal to 1, and a delta AIC of 0. Following the adjustment of these parameter configurations, the maximum number of iterations was established at 1000 with 10,000 background points. Additionally, 70% of the presence points were allocated for training the model, while the remaining data was reserved for testing purposes (Barbet‐Massin et al., 2012). However, the accuracy of this Maxent only model (AUC‐0.90, TSS‐0.82) was less than the model accuracy obtained from Ensemble model (AUC‐0.98, TSS‐0.966). Therefore, we decided to keep the Ensemble model prediction to generate our results and discussion.

## 3 RESULTS

### 3.1 Predicting the current suitable habitat of HMD

**FIGURE 2:**
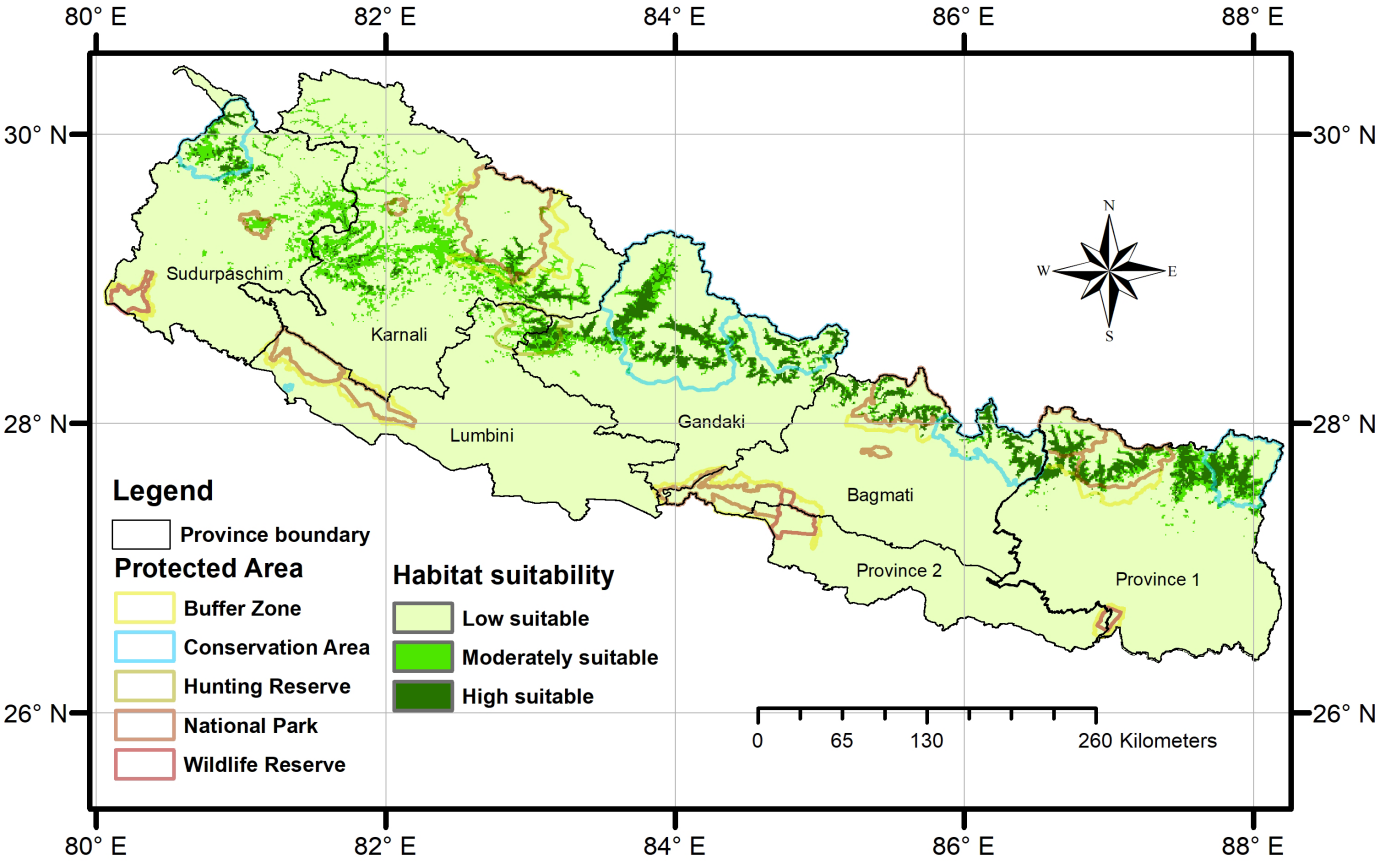
Present suitable habitat area for HMD (represented by green colors) across Nepal.

The current habitat suitability map generated through an ensemble modeling approach based on bioclimatic, topographic, vegetation, and anthropogenic variables indicated that 6973.76 km^2^ (5%) area of Nepal is highly suitable and 8387.11 km^2^ (6%) is moderately suitable for HMD (Figure 2).

Within the overall suitable habitat of HMD, approximately 51.4% (7895.88 km^2^) is located within protected areas of Nepal, while the remaining 48.6% exists outside the protected area networks. Among the protected areas of Nepal, Khaptad National Park has the most suitable habitat (54.37%) followed by Dhorpatan Hunting Reserve (51.12%), while the Sagarmatha NP and Shey‐Phoksundo NP has more suitable habitat in the buffer zones than inside the core area (Table 2). According to the administrative divisions, Karnali Province (3832.88 km^2^) has the most suitable area outside protected area networks followed by Sudurpaschim province (1184.6 km^2^) (Figure 2; Table 3).

**TABLE 2:** Current HMD suitable habitat within protected areas of Nepal.

Name of protected area	Total area (km^2^)	Highly suitable area (km^2^)	Moderately suitable area (km^2^)	Total suitable area (km^2^)	Suitable area (%)
Khaptad National Park	225	1.59	120.74	122.33	54.37
Dhorpatan Hunting Reserve	1325	308.1	369.33	677.46	51.12
Makalu Barun National Park	1500	483.07	199.58	682.66	45.51
Makalu Barun Buffer Zone	830	21.00	14.77	35.77	3.31
Kangchenjung Conservation Area	2035	567.19	314.81	882.00	43.34
Api Nampa Conservation Area	1903	331.85	358.46	690.32	36.28
Langtang National Park	1710	386.98	110.91	497.88	29.12
Langtang Buffer Zone	420	18.64	1.74	20.374	4.85
Gaurishankar Conservation Area	2179	544.91	93.45	638.36	29.30
Manaslu Conservation Area	1663	338.06	105.89	443.95	26.70
Annapurna Conservation Area	7629	1393.16	680.38	2073.54	27.18
Sagarmatha National Park	1148	231.14	61.46	292.60	25.49
Sagarmatha Buffer Zone	275	101.48	20.31	121.80	44.29
Rara National Park	106	0	28.13	28.13	26.55
Shey Phoksundo National Park	3555	144.00	200.88	344.89	9.7
Shey Phoksundo Buffer Zone	1349	38.20	305.57	343.77	25.48
Total		4909.44	2986.43	7895.88	

**TABLE 3:** Overall suitable area by province, along with the total suitable area that falls outside the protected area network.

Province name	Highly suitable area (km^2^)	Moderately suitable area (km^2^)	Total suitable area (km^2^)	The total suitable area outside PA (km^2^)
Province 1	2034.79	1055.20	3089.99	1075.14
Bagmati	1122.60	258.02	1380.63	224.01
Gandaki	2512.38	1407.36	3919.74	940.56
Lumbini	134.01	289.56	423.57	67.2
Karnali	695.72	3853.97	4549.69	3832.88
Sudurpaschim	474.26	1522.99	1997.25	1184.6
Madhesh	0	0	0	0
	6973.76	8387.11	15,360.87	7324.39

### 3.2 Contributions of variables to build the model

Among the 16 predictive environmental variables used to predict current suitable habitat for HMD, climatic variables had greater impacts that described more than 90% of the model performance. This model depicts Precipitation Seasonality (Bio15; 67%), followed by Precipitation of the Warmest Quarter (Bio18; 14%) as the major influencer for HMD distribution. Besides, the distribution of suitable habitats in the model was also influenced by the temperature ranges (both annual and diurnal) and distance to water bodies. And, among the anthropogenic variables, land use land cover change (LULC) had a major persuading factor in the model performance (Table 4).

**TABLE 4:** Percentage contribution of environmental variables to build the model.

Environmental variables	GBM	MAXENT.Phillips.2	RF	Percentage contribution
Slope	0	0.024	0.003	<1%
Aspect	0.001	0.004	0.003	<1%
Bio2	0.035	0.03	0.006	2%
Bio3	0.077	0.001	0.084	5%
Bio14	0.024	0.015	0.032	2%
Bio15	0.789	0.905	0.319	67%
Bio18	0.07	0.302	0.043	14%
Bio19	0.002	0.016	0.007	<1%
Distance to path	0.007	0.014	0.006	<1%
Distance to the motor road	0	0.013	0.004	<1%
Distance to settlement	0.002	0.003	0.014	<1%
Distance to water	0.061	0.052	0.024	5%
Forest	0.004	0.02	0.02	<1%
Population density	0.006	0	0.028	<1%
Livestock density	0.003	0	0.019	<1%
LULC	0	0.046	0	2%

**FIGURE 3:**
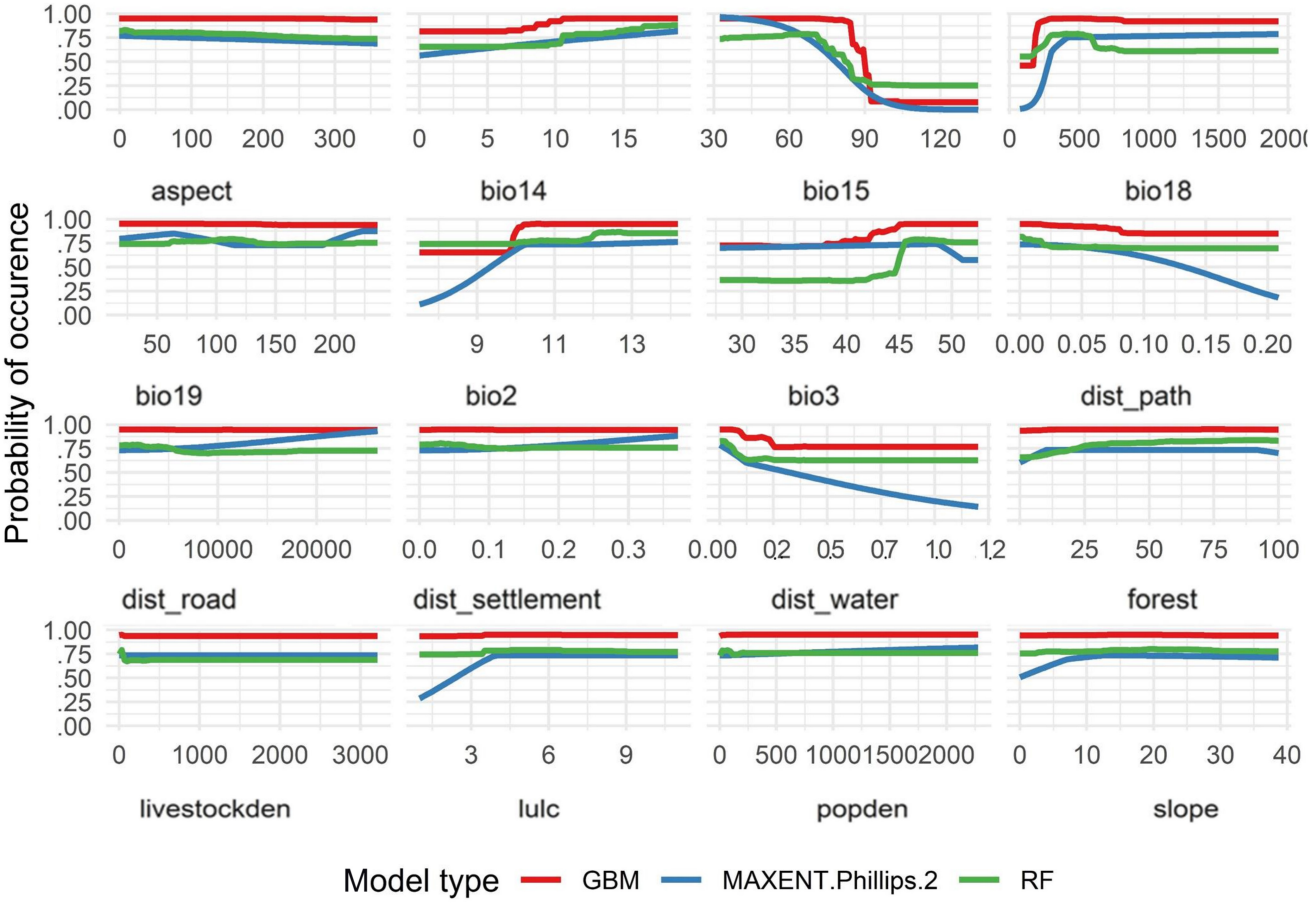
Response curves indicating the effects of different environmental variables on habitat suitability of HMD.

The response curve of the models indicates that the probability of occurrence of HMD decreases with an increase in Precipitation Seasonality (Bio15) and remains constant with an increase in Precipitation of the Warmest Quarter (Bio18) (Figure 3). Similarly, the probability of occurrence of HMD in habitats far from water sources decreases continuously under Maxent while remaining constant after a certain distance under GBM and RF model. The probability of occurrence of HMD is less in habitats with low forest cover but the probability of occurrence remains constant with further increase in forest cover.

## 4 DISCUSSION

### 4.1 Potentially suitable habitat for HMD

Our results indicate that suitable habitat for HMD is distributed throughout the mountainous regions of Nepal but is not thoroughly continuous, which aligns with previous studies conducted in the Nepalese Himalayas (Green, 1986). HMD has been recorded from all the Himalayan protected areas of Nepal (Aryal & Subedi, 2011). Our model showed that 51.78% (8295 km^2^) of the suitable habitat of musk deer is protected by the existing network of protected areas, which is in line with Lamsal et al. (2018), but contradicts with the prediction of Aryal and Subedi (2011), who suggested that only 19.26% (5815.08 km^2^) of potential habitat lies within protected areas. However, the total potential habitat of HMD predicted by Aryal and Subedi (2011) is much larger than the 16,020 km^2^ suitable habitat estimated in our study. Our model also revealed that 48.22% of suitable habitat lies outside protected areas, and the suitable habitat is not in a range throughout the mountainous regions which sustain the previous studies (Khadka et al., 2017; Lamsal et al., 2018; Singh, Gautam, et al., 2020; Singh, Mainali, et al., 2020).

Thus, we evaluated that identifying new areas outside PA networks is important for the conservation of HMD. Habitat distribution models provide bases to choose these areas of probable distribution of the species. Although HMD is one of the charismatic species of the Himalayas and is protected species under DNPWC Act, its studies and conservation actions are limited within the protected area. We found that almost 50% of the probable habitat of HMD lies outside the protected area networks, thus it is of immense need to expand the focus of conservation actions beyond the Pas too. Similarly, the suitable habitat outside PAs is larger in the western landscape compared to the central‐eastern complex. HMD is one of the most poached species (Ilyas, 2015; Subedi et al., 2012). Without prompt conservation actions and efforts beyond PAs networks, their survival will be increasingly confined to protected areas alone in the foreseeable future. Additionally, future climatically suitable habitat is predicted to be more in the western landscape of Nepal (Khadka et al., 2017). Although a good share of habitat lies within DHR and ANCA, research works are mere in these PAs compared to others. Therefore, the areas in the western landscape of Nepal require serious attention for the survival and conservation of HMD.

### 4.2 Distribution of HMD

Various factors such as climate, habitat, and anthropogenic variables influence the distribution of musk deer (Jiang et al., 2019; Singh, Gautam, et al., 2020; Singh, Mainali, et al., 2020; Wangdi et al., 2019). Our study's model predicted that climatic variables have a greater impact on the distribution of HMD in Nepalese Himalaya compared to habitat variables, which aligns with previous studies by Singh, Gautam, et al. (2020), Singh, Mainali, et al. (2020), and Lamsal et al. (2018). Precipitation is the most significant factor in determining the habitat suitability of HMD in Nepal Himalayas, which supports the findings of Singh, Gautam, et al. (2020) and Singh, Mainali, et al. (2020) but contradicts with findings of Lamsal et al. (2018) and Khadka et al. (2017). Specifically, our model predicted that HMD suitable habitat increases with an increase in precipitation during the warmest quarter, which is in line with research on other musk deer species (Singh, Gautam, et al., 2020; Singh, Mainali, et al., 2020). The model also suggested that the habitat suitability is influenced by precipitation in the dry winter and warm summer seasons, with a higher probability of occurrence of HMD in areas with higher seasonal precipitation, respectively (Figure 3).

In Nepal, the precipitation in summer is governed by monsoon, which brings heavy rainfall in the eastern part of the country and less rainfall in western regions, and the winter is governed by western disturbances which bring precipitation in the form of snow from the western region (Kansakar et al., 2004; Talchabhadel et al., 2018). The growth of vegetation in high mountains is affected by soil moisture, which in turn is influenced by temperature and precipitation patterns in the area (Paudel & Andersen, 2013; Regmi et al., 2020). The suitability of the HMD habitat is closely related to vegetation (Nandy et al., 2020).

The variability of precipitation seasonality across the region plays a significant role in determining the habitat suitability of HMD, as predicted by the model and reported in earlier studies (Khadka et al., 2017; Lamsal et al., 2018). As a result, the model projected a larger area of highly suitable habitats in the central‐eastern landscape compared to the western landscape (as shown in Figure 2 and Table 3). Vegetation growth in the trans‐Himalayan range was found to have a positive relationship with precipitation only (Ale et al., 2018; Tiwari et al., 2017). Studies have also reported that precipitation on the leeward sides is very low (Talchabhadel et al., 2018), which might be one of the reasons why the model predicted most of the suitable habitats in the gullies of river valleys. Overall, Nepal's precipitation seasonality is important in determining the distribution of HMD. Besides precipitation, the model diagnosed a strong response with temperature variables in determining the probability of occurrence in Nepalese Himalaya as demonstrated by mean diurnal range (BIO 2) and iso‐thermality (BIO 3) being the third‐most influential variables to the model. This relationship is consistent with previous studies (Khadka et al., 2017; Lamsal et al., 2018).

The likelihood of HMD occurrence was also influenced by topographic variables. Firstly, the proximity to water sources negatively impacts the probability of detecting HMD, with the likelihood of occurrence decreasing as the distance from water sources increases. This finding is consistent with previous research that highlights the importance of water availability in determining musk deer distribution (Singh et al., 2018; Thapamagar et al., 2021). Secondly, our model also predicted that HMD prefers gentle slopes, which aligns with earlier studies that report an increase in the likelihood of musk deer occurrence with slopes up to 20°, followed by a slight decrease in probability beyond that point (Aryal et al., 2010; Neupane et al., 2021; Thapamagar et al., 2018). Figure 3 presents a visual representation of these findings.

Likewise, our model did not identify any meaningful relationship with anthropogenic factors such as distance to road, distance to settlement, livestock density, and population density. This may be due to the seasonal and temporary nature of settlements in the high Himalayas in Nepal, where musk deer habitat might overlap with these settlements (Nandy et al., 2020). However, the model depicted that the probability of musk deer occurrence decreases far away from foot trails (Figure 3).

### 4.3 Conservation implications

In contrast to previous approaches that used single or general modeling techniques, our ensemble model incorporates multiple modeling algorithms to accurately predict the probable distribution of the highly endangered HMD species. Consequently, the outcomes of this study carry substantial implications for government authorities and conservation managers, providing valuable insights to enhance species conservation through improved connectivity and conservation measures beyond protected area networks. Our model offers informed knowledge to guide the development of scientific evidence‐based conservation actions specifically tailored to this globally endangered species. For instance, our model identifies a significant portion of HMD's habitats located outside protected areas, suggesting that conservation managers and concerned government authorities should prioritize efforts to establish connectivity among these suitable habitats and implement conservation measures in areas beyond protected areas as well.

## 5 CONCLUSION

Our ensemble model delineated the current suitable habitat of HMD throughout Nepal and showed that climatic variables, mainly precipitation seasonality and precipitation of the warmest quarter highly influence the distribution. We also suggest using this model and research approach in similar other species for predicting their future habitat distribution. Besides habitats of HMD located within protected areas network, our research outputs suggest employing immediate conservation efforts and promoting the connectivity among the significant proportion of suitable habitats lying outside the protected areas, which are at severe risk due to anthropogenic activities. This potential habitat distribution outputs provide guidelines for further research and conservation actions on HMD throughout Nepal. Precise habitat surveys and other fine‐scale ecological studies can be performed in highly suitable areas that will assist to formulate management interventions for converting less suitable habitat areas into more suitable areas in future.

## AUTHOR CONTRIBUTIONS

**Bijaya Dhami:** Conceptualization (equal); data curation (equal); formal analysis (equal); project administration (equal); resources (equal); writing – original draft (equal); writing – review and editing (equal). **Nar Bahadur Chhetri:** Data curation (equal); methodology (equal); resources (equal); writing – original draft (equal). **Bijaya Neupane:** Conceptualization (equal); data curation (equal); formal analysis (equal); methodology (equal); writing – original draft (equal); writing – review and editing (equal). **Binaya Adhikari:** Conceptualization (equal); data curation (equal); formal analysis (equal); resources (equal); software (equal); writing – original draft (equal); writing – review and editing (equal). **Bijay Bashyal:** Data curation (equal); resources (equal); writing – original draft (equal); writing – review and editing (equal). **Tek Maraseni:** Conceptualization (equal); project administration (equal); resources (equal); supervision (equal); writing – original draft (equal); writing – review and editing (equal). **Tilak Thapamagar:** Data curation (equal); resources (equal); writing – original draft (equal); writing – review and editing (equal). **Yogesh Dhakal:** Conceptualization (equal); data curation (equal); formal analysis (equal); project administration (equal); resources (equal); writing – original draft (equal). **Aashish Tripathi:** Data curation (equal); methodology (equal); project administration (equal); resources (equal); writing – original draft (equal). **Narayan Prasad Koju:** Conceptualization (equal); resources (equal); writing – original draft (equal); writing – review and editing (equal).

## CONFLICT OF INTEREST STATEMENT

None declared.

### OPEN RESEARCH BADGES

This article has earned Open Data, Open Materials and Preregistered Research Design badges. Data, materials and the preregistered design and analysis plan are available at https://doi.org/10.5061/dryad.sj3tx969j.

## Data Availability

The data associated with this manuscript are available at https://datadryad.org/stash/share/lFTZjuSKwcaLlaGKQkmIAotrQ1caH62_HZxgZJ1_60M.

## References

[ece310949-bib-0001] Adhikari, D. , Tiwary, R. , Singh, P. P. , Upadhaya, K. , Singh, B. , Haridasan, K. E. , Bhatt, B. B. , Chettri, A. , & Barik, S. K. (2019). Ecological niche modeling as a cumulative environmental impact assessment tool for biodiversity assessment and conservation planning: A case study of critically endangered plant *Lagerstroemia minuticarpa* in the Indian eastern Himalaya. Journal of Environmental Management, 243, 299–307. 10.1016/j.jenvman.2019.05.036 31102897

[ece310949-bib-0002] Aiello‐Lammens, M. E. , Boria, R. A. , Radosavljevic, A. , Vilela, B. , & Anderson, R. P. (2015). spThin: An R package for spatial thinning of species occurrence records for use in ecological niche models. Ecography (Cop.), 38, 541–545. 10.1111/ecog.01132

[ece310949-bib-0003] Ale, R. , Zhang, L. , Li, X. , Raskoti, B. B. , & Pugnaire, F. I. (2018). Leaf δ13C as an indicator of water availability along elevation gradients in the dry Himalayas. Ecological Indicator, 94, 266–273. 10.1016/j.ecolind.2018.07.002

[ece310949-bib-0004] Allouche, O. , Tsoar, A. , & Kadmon, R. (2006). Assessing the accuracy of species distribution models: Prevalence, kappa and the true skill statistic (TSS). Journal of Applied Ecology, 43, 1223–1232. 10.1111/j.1365-2664.2006.01214.x

[ece310949-bib-0005] Amin, R. , Baral, H. S. , Lamichhane, B. R. , Poudyal, L. P. , Lee, S. , Jnawali, S. R. , Acharya, K. P. , Upadhyaya, G. P. , Pandey, M. B. , & Shrestha, R. (2018). The status of Nepal's mammals. Journal of Threatened Taxa, 10, 11361–11378. 10.11609/jott.3712.10.3.11361-11378

[ece310949-bib-0006] Araújo, M. B. , & Guisan, A. (2006). Five (or so) challenges for species distribution modelling. Journal of Biogeography, 33(10), 1677–1688. 10.1111/j.1365-2699.2006.01584.x

[ece310949-bib-0008] Aryal, A. , Raubenheimer, D. , Subedi, S. , & Kattel, B. (2010). Spatial habitat overlap and habitat preference of Himalayan musk deer (“*Moschus chrysogaster*”) in Sagarmatha (Mt. Everest) National Park, Nepal. Current Research Journal of Biological Sciences, 2(3), 217–225. https://www.airitilibrary.com/Publication/alDetailedMesh?DocID=20410778‐201005‐201512220011‐201512220011‐217‐225

[ece310949-bib-0009] Aryal, A. , Shrestha, U. B. , Ji, W. , Ale, S. B. , Shrestha, S. , Ingty, T. , Maraseni, T. , Cockfield, G. , & Raubenheimer, D. (2016). Predicting the distributions of predator (snow leopard) and prey (blue sheep) under climate change in the Himalaya. Ecology and Evolution, 6, 4065–4075. 10.1002/ece3.2196 27516864 PMC4875782

[ece310949-bib-0010] Aryal, A. , & Subedi, A. (2011). The conservation and potential habitat of the Himalayan musk deer, *Moschus chrysogaster*, in the protected areas of Nepal. International Journal of Conservation Science, 2(2), 81–89.

[ece310949-bib-0012] Barbet‐Massin, M. , Jiguet, F. , Albert, C. H. , & Thuiller, W. (2012). Selecting pseudo‐absences for species distribution models: How, where and how many? Methods in Ecology and Evolution, 3, 327–338. 10.1111/j.2041-210X.2011.00172.x

[ece310949-bib-0013] Beery, S. , Cole, E. , Parker, J. , Perona, P. , Winner, K. , 2021. Species distribution modeling for machine learning practitioners: A review. In: ACM SIGCAS Conference on Computing and Sustainable Societies (329–348). 10.1145/3460112.3471966

[ece310949-bib-0014] Bhattacharjee, A. , Anadón, J. D. , Lohman, D. J. , Doleck, T. , Lakhankar, T. , Shrestha, B. B. , Thapa, P. , Devkota, D. , Tiwari, S. , Jha, A. , & Siwakoti, M. (2017). The impact of climate change on biodiversity in Nepal: Current knowledge, lacunae, and opportunities. Climate, 5(4), 80. 10.3390/cli5040080

[ece310949-bib-0015] Boria, R. A. , Olson, L. E. , Goodman, S. M. , & Anderson, R. P. (2014). Spatial filtering to reduce sampling bias can improve the performance of ecological niche models. Ecological Modelling, 275, 73–77. 10.1016/j.ecolmodel.2013.12.012

[ece310949-bib-0016] Chhetri, P. K. , & Cairns, D. M. (2015). Contemporary and historic population structure of *Abies spectabilis* at treeline in Barun valley, Eastern Nepal Himalaya. Journal of Mountain Science, 12, 558–570. 10.1007/s11629-015-3454-5

[ece310949-bib-0018] Dhami, B. , Adhikari, B. , Panthi, S. , & Neupane, B. (2023). Predicting suitable habitat of swamp deer (*Rucervus duvaucelii*) across the Western Terai arc landscape of Nepal. Heliyon, 9(6), e16639. 10.1016/j.heliyon.2023.e16639 37274642 PMC10238933

[ece310949-bib-0019] DNPWC . (2013). Protected area of Nepal [Online]. Nepal (http://www.dnpwc.gov.np): DNPWC. http://www.dnpwc.gov.np [Accessed February 2 2014].

[ece310949-bib-0020] Dormann, C. F. , Elith, J. , Bacher, S. , Buchmann, C. , Carl, G. , Carré, G. , Marquéz, J. R. G. , Gruber, B. , Lafourcade, B. , & Leitão, P. J. (2013). Collinearity: A review of methods to deal with it and a simulation study evaluating their performance. Ecography (Cop.)., 36, 27–46. 10.1111/j.1600-0587.2012.07348.x

[ece310949-bib-0021] Elith, J. , & Leathwick, J. R. (2009). Species distribution models: Ecological explanation and prediction across space and time. Annual Review of Ecology, Evolution, and Systematics, 40, 677–697. 10.1146/annurev.ecolsys.110308.120159

[ece310949-bib-0022] Elith, J. , Phillips, S. J. , Hastie, T. , Dudík, M. , Chee, Y. E. , & Yates, C. J. (2011). A statistical explanation of MaxEnt for ecologists. Diversity and Distributions, 17(1), 43–57. 10.1111/j.1472-4642.2010.00725.x

[ece310949-bib-0023] Elsen, P. R. , Monahan, W. B. , & Merenlender, A. M. (2020). Topography and human pressure in mountain ranges alter expected species responses to climate change. Nature Communications, 11, 1–10. 10.1038/s41467-020-15881-x PMC7181879 32332913

[ece310949-bib-0024] Elsen, P. R. , Tingley, M. W. , Kalyanaraman, R. , Ramesh, K. , & Wilcove, D. S. (2017). The role of competition, ecotones, and temperature in the elevational distribution of Himalayan birds. Ecology, 98, 337–348. 10.1002/ecy.1669 27869987

[ece310949-bib-0025] ESRI . (2020). ArcGIS Desktop: Release 10.8.1. Environmental Systems Research Institute.

[ece310949-bib-0026] Fick, S. E. , & Hijmans, R. J. (2017). WorldClim 2: New 1‐km spatial resolution climate surfaces for global land areas. International Journal of Climatology, 37, 4302–4315. 10.1002/joc.5086

[ece310949-bib-0027] Forester, B. R. , DeChaine, E. G. , & Bunn, A. G. (2013). Integrating ensemble species distribution modelling and statistical phylogeography to inform projections of climate change impacts on species distributions. Diversity and Distributions, 19, 1480–1495. 10.1111/ddi.12098

[ece310949-bib-0028] Forrest, J. L. , Wikramanayake, E. , Shrestha, R. , Areendran, G. , Gyeltshen, K. , Maheshwari, A. , Mazumdar, S. , Naidoo, R. , Thapa, G. J. , & Thapa, K. (2012). Conservation and climate change: Assessing the vulnerability of snow leopard habitat to treeline shift in the Himalaya. Biological Conservation, 150, 129–135. 10.1016/j.biocon.2012.03.001

[ece310949-bib-0029] Gandiwa, E. (2014). Vegetation factors influencing density and distribution of wild large herbivores in a Southern African Savannah. African Journal of Ecology, 52, 274–283. 10.1111/aje.12114

[ece310949-bib-0030] GEOFABRIK . (2022). Open Street Map Data in Layered GIS Format Version 0.7.12‐2022‐03‐31. http://download.geofabrik.de/

[ece310949-bib-0031] Ghimire, S. K. , Dhamala, M. K. , Lamichhane, B. R. , Ranabhat, R. , Khim Bahadur, K. C. , & Poudel, S. (2019). Identification of suitable habitat for swamp deer *Rucervus duvaucelii* duvaucelii (Mammalia: Artiodactyla: Cervidae) in Chitwan National Park, Nepal. Journal of Threatened Taxa, 11, 13644–13653. 10.11609/jott.4129.11.6.13644-13653

[ece310949-bib-0032] Green, M. J. B. (1986). The distribution, status and conservation of the Himalayan musk deer *Moschus chrysogaster* . Biological Conservation, 35(4), 347–375. 10.1016/00063207(86)90094-7

[ece310949-bib-0034] Hansen, M. C. , Potapov, P. V. , Moore, R. , Hancher, M. , Turubanova, S. A. , Tyukavina, A. , Thau, D. , Stehman, S. V. , Goetz, S. J. , & Loveland, T. R. (2013). High‐resolution global maps of 21st‐century forest cover change. Science, 342, 850–853. 10.1126/science.1244693 24233722

[ece310949-bib-0035] Hao, T. , Elith, J. , Guillera‐Arroita, G. , & Lahoz‐Monfort, J. J. (2019). A review of evidence about use and performance of species distribution modelling ensembles like BIOMOD. Diversity and Distributions, 25, 839–852. 10.1111/ddi.12892

[ece310949-bib-0036] Hao, T. , Elith, J. , Lahoz‐Monfort, J. J. , & Guillera‐Arroita, G. (2020). Testing whether ensemble modelling is advantageous for maximising predictive performance of species distribution models. Ecography (Cop.)., 43, 549–558. 10.1111/ecog.04890

[ece310949-bib-0037] Harris, R. (2016). *Moschus chrysogaster*. Alp. Musk Deer, IUCN red List 197.

[ece310949-bib-0038] HDX . (2022). Search for a dataset humanitarian data exchange. HDX. https://data.humdata.org/dataset

[ece310949-bib-0039] Hijmans, R. J. (2012). Cross‐validation of species distribution models: Removing spatial sorting bias and calibration with a null model. Ecology, 93, 679–688. 10.1890/11-0826.1 22624221

[ece310949-bib-0040] Hijmans, R. J. , Cameron, S. E. , Parra, J. L. , Jones, P. G. , & Jarvis, A. (2005). Very high resolution interpolated climate surfaces for global land areas. International Journal of Climatology, 25, 1965–1978. 10.1002/joc.1276

[ece310949-bib-0041] ICIMOD . (2022). International Centre for Integrated Mountain Development (Regional Database System). Land cover of Nepal. http://rds.icimod.org

[ece310949-bib-0042] Ilyas, O. (2015). Status, habitat use and conservation of alpine musk deer (*Moschus chrysogaster*) in Uttarakhand Himalayas, India. Journal of Applied Animal Research, 43, 83–91. 10.1080/09712119.2014.899495

[ece310949-bib-0044] Jiang, F. , Zhang, J. , Gao, H. , Cai, Z. , Zhou, X. , Li, S. , & Zhang, T. (2019). Musk deer (*Moschus* spp.) face redistribution to higher elevations and latitudes under climate change in China. The Science of the Total Environment, 704, 135335. 10.1016/j.scitotenv.2019.135335 31784177

[ece310949-bib-0045] Jnawali, S. , Baral, H. , Lee, S. , Acharya, K. , Upadhyay, G. , Pandey, M. , Shrestha, R. , Joshi, D. , Lamichhane, B. , & Griffiths, J. (2011). The Status of Nepal's Mammals: The National Red List Series‐IUCN. Department of National Parks and Wildlife Conservation, Kathmandu, Nepal, 276. 10.13140/RG.2.1.2561.7448

[ece310949-bib-0046] Jönsson, P. , & Eklundh, L. (2004). TIMESAT—A program for analyzing time‐series of satellite sensor data. Computational Geosciences, 30, 833–845. 10.1016/j.cageo.2004.05.006

[ece310949-bib-0047] Kansakar, S. R. , Hannah, D. M. , Gerrard, J. , & Rees, G. (2004). Spatial pattern in the precipitation regime of Nepal. International Journal of Climatology, 24, 1645–1659. 10.1002/joc.1098

[ece310949-bib-0048] Karki, R. , Talchabhadel, R. , Aalto, J. , & Baidya, S. K. (2016). New climatic classification of Nepal. Theoretical and Applied Climatology, 125, 799–808. 10.1007/s00704-015-1549-0

[ece310949-bib-0049] Kass, J. M. , Muscarella, R. , Galante, P. J. , Bohl, C. L. , Pinilla‐Buitrago, G. E. , Boria, R. A. , Soley‐Guardia, M. , & Anderson, R. P. (2021). ENMeval 2.0: Redesigned for customizable and reproducible modeling of species' niches and distributions. Methods in Ecology and Evolution, 12(9), 1602–1608. 10.1111/2041-210X.13628

[ece310949-bib-0050] Khadka, K. K. , Kannan, R. , Ilyas, O. , Abbas, F. I. , & James, D. A. (2017). Where are they? Where will they be? In pursuit of current and future whereabouts of endangered Himalayan musk deer. Mammalian Biology, 85, 30–36. 10.1016/j.mambio.2017.02.007

[ece310949-bib-0051] Koju, N. , Chalise, M. , & Kyes, R. (2021). Acute cold stress: A potential threat to Royle's pika (*Ochotona roylii*) survival at Central Himalayas of Nepal. Banko Janakari, 31, 33–40. 10.3126/BANKO.V31I1.37343

[ece310949-bib-0052] Koju, N. P. , Bashyal, B. , & Shah, S. N. (2021). Habitat preference of Himalayan musk deer (*Moschus leucogaster* Hodgson, 1839) at Lapchi of Bigu rural municipality, Gaurishankar conservation area. Nepal Journal of Environmental Science, 9, 21–28. 10.3126/njes.v9i1.37844

[ece310949-bib-0053] Lamsal, P. , Kumar, L. , Aryal, A. , & Atreya, K. (2018). Future climate and habitat distribution of Himalayan musk deer (*Moschus chrysogaster*). Ecological Informatics, 44, 101–108. 10.1016/j.ecoinf.2018.02.004

[ece310949-bib-0054] Lobo, J. , Jiménez‐valverde, A. , & Real, R. (2008). AUC: Erratum: Predicting species distribution: Offering more than simple habitat models. Global Ecology and Biogeography, 17, 145–151. 10.1111/j.1461-0248.2005.00792.x

[ece310949-bib-0055] Manish, K. , Telwala, Y. , Nautiyal, D. C. , & Pandit, M. K. (2016). Modelling the impacts of future climate change on plant communities in the Himalaya: A case study from eastern Himalaya, India. Modeling Earth Systems and Environment, 2, 1–12. 10.1007/s40808-016-0163-1

[ece310949-bib-0056] Mao, M. , Chen, S. , Ke, Z. , Qian, Z. , & Xu, Y. (2022). Using MaxEnt to predict the potential distribution of the little fire ant (*Wasmannia auropunctata*) in China. Insects, 13(11), 1008. 10.3390/insects13111008 36354830 PMC9698453

[ece310949-bib-0057] Marmion, M. , Parviainen, M. , Luoto, M. , Heikkinen, R. K. , & Thuiller, W. (2009). Evaluation of consensus methods in predictive species distribution modelling. Diversity and Distributions, 15, 59–69. 10.1111/j.1472-4642.2008.00491.x

[ece310949-bib-0058] Nandy, S. , Neethu Lakshmi, M. , & Kushwaha, S. P. (2020). Habitat suitability analysis of Himalayan musk deer (*Moschus leucogaster*) in part of Western Himalaya, India. Journal of the Indian Society of Remote Sensing, 48, 1523–1533. 10.1007/s12524-020-01175-8

[ece310949-bib-0059] Neupane, B. , Chhetri, N. B. , & Dhami, B. (2021). Habitat selection of Himalayan musk deer *Moschus leucogaster* (Mammalia: Artiodactyla: Moschidae) with respect to biophysical attributes in Annapurna conservation area of Nepal. Journal of Threatened Taxa, 13, 18703–18712. 10.11609/jott.6725.13.7.18703-18712

[ece310949-bib-0060] ODN (Open Data Nepal) . (2022). Livestock Population (Ministry of Agricultural Development). https://opendatanepal.com/dataset/livestock‐population

[ece310949-bib-0100] Paudel, K. P. , & Andersen, P. (2013). Response of rangeland vegetation to snow cover dynamics in Nepal Trans Himalaya. Climatic Change, 117, 149–162. 10.1007/s10584-012-0562-x

[ece310949-bib-0061] Paudel, P. K. , Bhattarai, B. P. , & Kindlmann, P. (2012). An overview of the biodiversity in Nepal. In P. Kindlmann (Ed.), Himalayan biodiversity in the changing world (pp. 1–40). Springer. 10.1007/978-94-007-1802-9_1

[ece310949-bib-0062] Perea, R. , Perea‐García‐Calvo, R. , Díaz‐Ambrona, C. G. , & San Miguel, A. (2015). The reintroduction of a flagship ungulate *Capra pyrenaica*: Assessing sustainability by surveying woody vegetation. Biological Conservation, 181, 9–17. 10.1016/j.biocon.2014.10.018

[ece310949-bib-0063] Peterson, A. T. , & Soberón, J. (2012). Species distribution modeling and ecological niche modeling: Getting the concepts right. Nature Conservation, 10, 102–107. 10.4322/NATCON.2012.019

[ece310949-bib-0064] R Development Core Team . (2020). R: A language and environment for statistical computing. R Foundation for Statistical Computing. https://apps.dtic.mil/sti/pdfs/AD1039033.pdf

[ece310949-bib-0065] Raymond, C. V. , McCune, J. L. , Rosner‐Katz, H. , Chadès, I. , Schuster, R. , Gilbert, B. , & Bennett, J. R. (2020). Combining species distribution models and value of information analysis for spatial allocation of conservation resources. Journal of Applied Ecology, 57, 819–830. 10.1111/1365-2664.13580

[ece310949-bib-0066] Regmi, R. , Ma, Y. , Ma, W. , Baniya, B. , & Bashir, B. (2020). Interannual variation of NDVI, precipitation and temperature during the growing season in Langtang National Park, central Himalaya, Nepal. Applied Ecology and Environmental Sciences, 8(5), 218–228.

[ece310949-bib-0067] Renner, I. W. , & Warton, D. I. (2013). Equivalence of MAXENT and Poisson point process models for species distribution modeling in ecology. Biometrics, 69(1), 274–281. 10.1111/j.1541-0420.2012.01824.x 23379623

[ece310949-bib-0068] Sharma, P. , Panthi, S. , Yadav, S. K. , Bhatta, M. , Karki, A. , Duncan, T. , Poudel, M. , & Acharya, K. P. (2020). Suitable habitat of wild Asian elephant in Western Terai of Nepal. Ecology and Evolution, 10, 6112–6119. 10.1002/ece3.6356 32607217 PMC7319151

[ece310949-bib-0070] Shrestha, A. B. , & Aryal, R. (2011). Climate change in Nepal and its impact on Himalayan glaciers. Regional Environmental Change, 11, 65–77. 10.1007/s10113-010-0174-9

[ece310949-bib-0071] Shrestha, A. B. , Wake, C. P. , Dibb, J. E. , & Mayewski, P. A. (2000). Precipitation fluctuations in the Nepal Himalaya and its vicinity and relationship with some large scale climatological parameters. International Journal of Climatology, 20, 317–327. 10.1002/(SICI)1097-0088(20000315)20:3<317::AID-JOC476>3.0.CO;2-G

[ece310949-bib-0073] Shrestha, U. B. , Shrestha, S. , Chaudhary, P. , & Chaudhary, R. P. (2010). How representative is the protected areas system of Nepal? Mountain Research and Development, 30, 282–294. 10.1659/MRD-JOURNAL-D-10-00019.1

[ece310949-bib-0074] Singh, B. , Gautam, K. B. , Sahoo, S. , Kumar, A. , & Gupta, S. K. (2020). Complete mitogenome of Kashmir musk deer (*Moschus cupreus*) and its comparative phylogenetic relationships. Molecular Biology Reports, 47, 9757–9763. 10.1007/s11033-020-06002-4 33219479

[ece310949-bib-0075] Singh, P. B. , Mainali, K. , Jiang, Z. , Thapa, A. , Subedi, N. , Awan, M. N. , Ilyas, O. , Luitel, H. , Zhou, Z. , & Hu, H. (2020). Projected distribution and climate refugia of endangered Kashmir musk deer *Moschus cupreus* in greater Himalaya, South Asia. Scientific Reports, 10, 1511. 10.1038/s41598-020-58111-6 32001721 PMC6992763

[ece310949-bib-0076] Singh, P. B. , Shrestha, B. B. , Thapa, A. , Saud, P. , & Jiang, Z. (2018). Selection of latrine sites by Himalayan musk deer (*Moschus leucogaster*) in Neshyang Valley, Annapurna conservation area, Nepal. Journal of Applied Animal Research, 46, 920–926. 10.1080/09712119.2018.1430578

[ece310949-bib-0077] Steen, B. , Cardoso, A. C. , Tsiamis, K. , Nieto, K. , Engel, J. , & Gervasini, E. (2019). Modelling hot spot areas for the invasive alien plant *Elodea nuttallii* in the EU. Management of Biological Invasions, 10(1), 151–170. 10.3391/mbi.2019.10.1.10

[ece310949-bib-0079] Su, B. , Wang, Y. X. , Lan, H. , Wang, W. , & Zhang, Y. (1999). Phylogenetic study of complete cytochrome b genes in musk deer (genus Moschus) using museum samples. Molecular Phylogenetics and Evolution, 12(3), 241–249. 10.1006/mpev.1999.0616 10413620

[ece310949-bib-0080] Subedi, A. , Aryal, A. , Koirala, R. K. , Timilsina, Y. P. , Meng, X. , & McKenzie, F. (2012). Habitat ecology of Himalayan musk deer (*Moschus chrysogaster*) in Manaslu conservation area, Nepal. International Journal of Zoological Research, 8, 81–89. 10.3923/ijzr.2012.81.89

[ece310949-bib-0081] Syed, Z. , & Ilyas, O. (2015). Habitat preference and feeding ecology of alpine musk deer (*Moschus chrysogaster*) in Kedarnath wildlife sanctuary, Uttarakhand, India. Animal Production Science, 56, 978–987. 10.1071/AN141028

[ece310949-bib-0082] Talchabhadel, R. , Karki, R. , Thapa, B. R. , Maharjan, M. , & Parajuli, B. (2018). Spatio‐temporal variability of extreme precipitation in Nepal. International Journal of Climatology, 38, 4296–4313. 10.1002/joc.5669

[ece310949-bib-0083] Thapamagar, T. , Bhandari, S. , Acharya, H. R. , Awasthi, B. , Magar, K. T. , Bhusal, D. R. , & Youlatos, D. (2021). Habitat utilization by Alpine Musk Deer, *Moschus chrysogaster* (Artiodactyla, Moschidae), in Khaptad National Park, Nepal. Zoodiversity, 55, 405–410. 10.15407/zoo2021.05.405

[ece310949-bib-0084] Thapamagar, T. , Magar, K. T. , Pandey, M. , Bhandari, S. , & Bhusal, D. R. (2018). Habitat preferences and conservation status of Himalayan musk deer (*Moschus chrysogaster*) in Langtang National Park, Nepal. Journal of Ecology and Natural Resources, 2(6), 000152. 10.23880/jenr-16000152

[ece310949-bib-0085] Thuiller, W. (2004). Patterns and uncertainties of species' range shifts under climate change. Global Change Biology, 10(12), 2020–2027. 10.1111/gcb.12727 PMC4340562 25200514

[ece310949-bib-0086] Thuiller, W. , Georges, D. , Engler, R. , & Breiner, F. (2020). biomod2: Ensemble platform for species distribution modeling. R package version 3.4.6. https://cran.r‐project.org/web/packages/biomod2/index.html

[ece310949-bib-0087] Thuiller, W. , Lafourcade, B. , Engler, R. , & Araújo, M. B. (2009). BIOMOD–a platform for ensemble forecasting of species distributions. Ecography (Cop.)., 32, 369–373. 10.1111/j.1600-0587.2008.05742.x

[ece310949-bib-0088] Timmins, R. J. , & Duckworth, J. W. (2015). “*Moschus leucogaster*”. *IUCN Red List of Threatened Species* . **2015**:e.T13901A61977764 doi, 10.2305/IUCN.UK.20152.RLTS.T13901A61977764.en

[ece310949-bib-0089] Tiwari, A. , Fan, Z. X. , Jump, A. S. , Li, S. F. , & Zhou, Z. K. (2017). Gradual expansion of moisture sensitive Abies spectabilis forest in the trans‐Himalayan zone of central Nepal associated with climate change. Dendrochronologia, 41, 34–43. 10.1016/j.dendro.2016.01.006

[ece310949-bib-0090] USGS . (2022). Earth Explorer. https://earthexplorer.usgs.gov/

[ece310949-bib-0091] Van Gils, J. A. , Lisovski, S. , Lok, T. , Meissner, W. , Ożarowska, A. , De Fouw, J. , Rakhimberdiev, E. , Soloviev, M. Y. , Piersma, T. , & Klaassen, M. (2016). Body shrinkage due to Arctic warming reduces red knot fitness in tropical wintering range. Science, 352, 819–821. 10.1126/science.aad6351 27174985

[ece310949-bib-0092] Veloz, S. D. (2009). Spatially autocorrelated sampling falsely inflates measures of accuracy for presence‐only niche models. Journal of Biogeography, 36, 2290–2299. 10.1111/j.1365-2699.2009.02174.x

[ece310949-bib-0099] Wangdi, T. , Tobgay, S. , Dorjee, K. , Dorji, K. , & Wangyel, S. (2019). The distribution, status and conservation of the Himalayan Musk Deer *Moschus chrysogaster* in Sakteng Wildlife Sanctuary. Global Ecology and Conservation, 17, e00466. 10.1016/j.gecco.2018.e00466

[ece310949-bib-0095] Xiuxiang, M. , Caiquan, Z. , Jinchu, H. , Cao, L. , Zhibin, M. , Jinchao, F. , Yijun, Z. , & Yinjiu, Z. (2006). Musk deer farming in China. Animal Science, 82, 1–6. 10.1079/ASC200516

[ece310949-bib-0097] Zeng, Y. , Low, B. W. , & Yeo, D. C. J. (2016). Novel methods to select environmental variables in MaxEnt: A case study using invasive crayfish. Ecological Modelling, 341, 5–13. 10.1016/j.ecolmodel.2016.09.019

[ece310949-bib-0098] Zurell, D. , Franklin, J. , König, C. , Bouchet, P. J. , Dormann, C. F. , Elith, J. , Fandos, G. , Feng, X. , Guillera‐Arroita, G. , & Guisan, A. (2020). A standard protocol for reporting species distribution models. Ecography (Cop.)., 43, 1261–1277. 10.1111/ecog.04960

